# Comparison of 12-Month Outcomes with Zotarolimus- and Paclitaxel-Eluting Stents: A Meta-Analysis

**DOI:** 10.5402/2011/675638

**Published:** 2011-05-26

**Authors:** Rohit S. Loomba, Suraj Chandrasekar, Neil Malhotra, Rohit R. Arora

**Affiliations:** ^1^Department of Cardiology, James A Lovell Federal Health Center/Chicago Medical School, 3001 Green Bay Road, North Chicago, IL 60064, USA; ^2^Department of Medicine, Saint Louis University Medical School, 1402 South Grand, St. Louis, MO 63104, USA

## Abstract

Revascularization after myocardial infarction is often achieved via percutaneous coronary intervention, which often entails stenting. Drug-eluting stents have shown benefits over bare metal stents in this setting, and a variety of drug-eluting stents are now available, including sirolimus-, paclitaxel-, and zotarolimus-eluting stents. There are studies that have compared the various drug-eluting stents and this meta-analysis pools data comparing 12-month clinical outcomes of zotarolimus- and paclitaxel-eluting stents. End points studied were myocardial infarction, major adverse cardiac events, cardiac death, all-cause death, stent thrombosis, target vessel revascularization, and target lesion revascularization.There was a statistically significant reduction in risk of myocardial infarction (odds ratio, 0.250, confidence interval, 0.160 to 0.392) and statistically insignificant reductions in major adverse cardiac events (odds ratio, 0.813, confidence interval, 0.656 to 1.007), cardiac death (odds ratio, 0.817, confidence interval, 0.359 to 1.857), all cause death (odds ratio, 0.820, confidence interval, 0.443 to 1.516), and target lesion revascularization (odds ratio, 0.936, confidence interval 0.702 to 1.247). There was a statistically significant increase in target vessel revascularization (odds ratio, 1.336, confidence interval, 1.003 to 1.778) and a statistically insignificant increase in stent thrombosis (odds ratio, 1.174, confidence interval, 0.604 to 2.280). These findings are similar to the individual studies although other studies have noted increased late loss with zotarolimus-eluting stents and this current data associated with late loss should be kept in mind when makimg clinical decisions regarding sent selection.

## 1. Introduction

Management of coronary disease has evolved immensely over the past 40 years. Coronary artery bypass grafting (CABG) has become less common with the development of percutaneous coronary intervention (PCI). PCI has now become standard of care for managing patients with myocardial infarction (MI), with stenting techniques replacing balloon angioplasty. The increased role of stenting has led to the development of drug-eluting stents which have been shown to lower restenosis rates when compared to bare metal stents, without increasing the risk of MI or death [1]. There are various options when selecting drug-eluting stents, including paclitaxel-, sirolimus, and zotarolimus-eluting stents. With several options when it comes to drug-eluting stents, the need for evidence-based guidelines has become evident. This meta-analysis pools data from studies comparing 12-month clinical outcomes of newer zotarolimus-eluting stents to commonly used paclitaxel-eluting stents.

## 2. Methods

### 2.1. Literature Sources, Search Terms, and Study Selection

**Figure 1:**
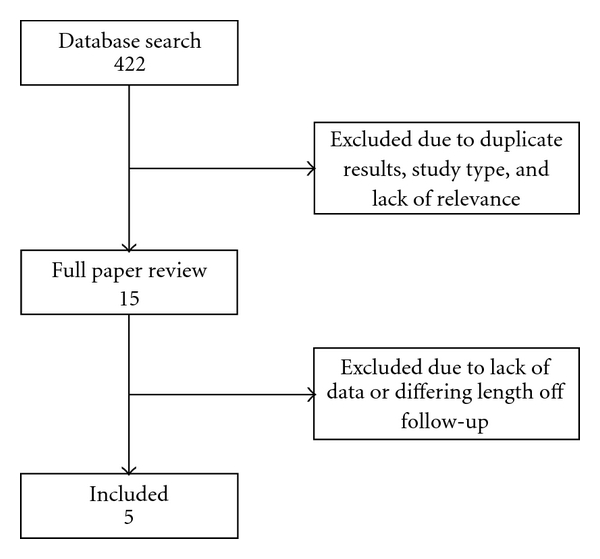
Overview of study selection.

Systematic review of medical literature was carried out to identify studies evaluating outcomes after stenting with zotarolimus- and paclitaxel-eluting stents. Studies were collected by searching MEDLINE and the Cochrane Library using web-based search engines such as OVID. All relevant studies were assessed for inclusion regardless of time of publication. Search terms used include zotarolimus, paclitaxel, drug-eluting stents, stent thrombosis, stent outcomes, and combinations of these terms. Hand search for articles, abstracts, and reviews was also conducted using references of already identified studies. Explicit inclusion and exclusion criteria were used to evaluate the titles and abstracts from collected articles on basis of the aforementioned criteria for potential inclusion.  Figure 1 outlines study selection.

### 2.2. End Points and Definitions

A total of seven end points were extracted from five studies [2–6]. End points studied were myocardial infarction (MI), major adverse cardiac events (MACE), cardiac death, all-cause death, stent thrombosis, target vessel revascularization, and target lesion revascularization. Only studies with corresponding endpoint definitions were included.

### 2.3. Data Extraction and Quality Assessment

After articles were collected and screened for inclusion, full articles were retrieved for titles thought to fulfill inclusion criteria. Data was then extracted while also scoring the methodological quality of each study.

### 2.4. Statistical Analysis

Individual patient data from included studies was not available, so a meta-analysis was done using summary statistics from each. Statistical analysis was performed using the MedCalc software package (Version 11.3, Mariakerke, Belgium). Cochrane's Q statistics were calculated and used to determine the heterogeneity of the studies for each end point. The end points demonstrated homogeneous results so the fixed effects model was used for analysis (Table 1). A two-sided alpha error less than 0.05 was considered to be statistically significant. Heterogeneity analysis is summarized in Table 1.

## 3. Results

### 3.1. Baseline Characteristics

**Figure 2:**
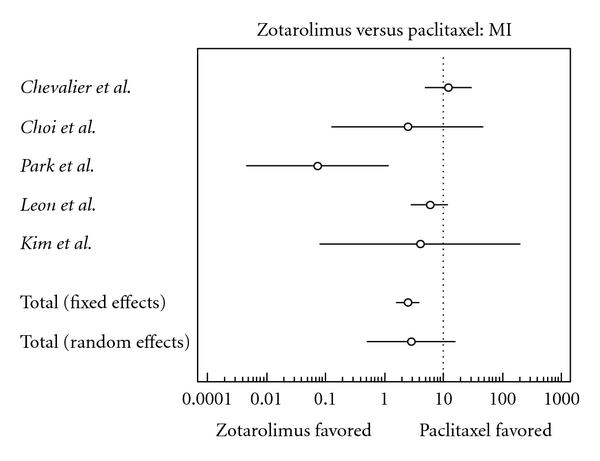
Forest plot comparing risk of myocardial infarction.

**Figure 3:**
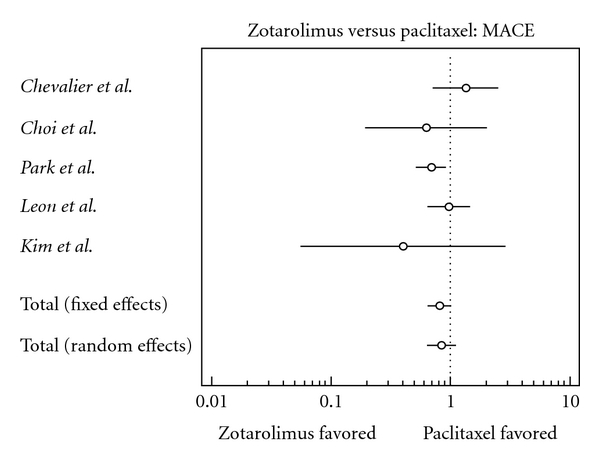
Forest plot comparing risk of major adverse cardiac events.

**Figure 4:**
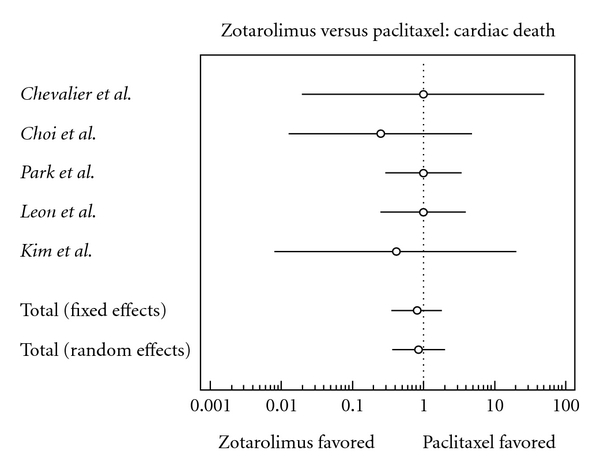
Forest plot comparing risk of cardiac death.

**Figure 5:**
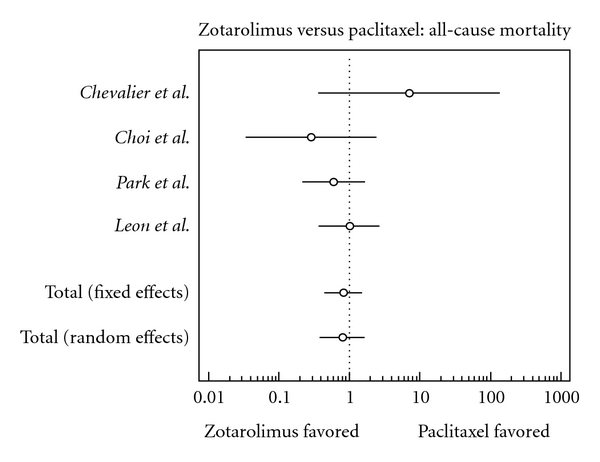
Forest plot comparing risk of all-cause mortality.

**Figure 6:**
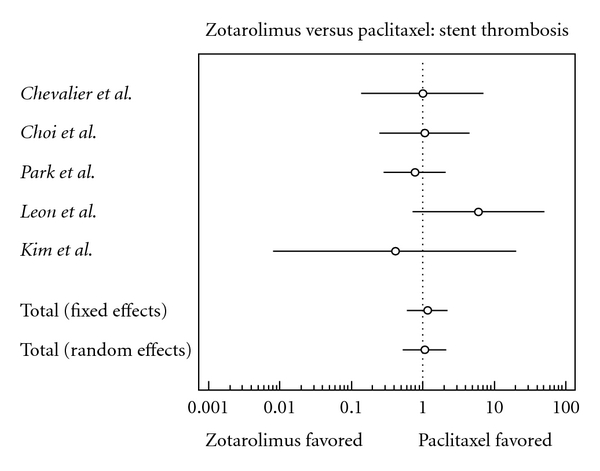
Forest plot comparing risk of stent thrombosis.

**Figure 7:**
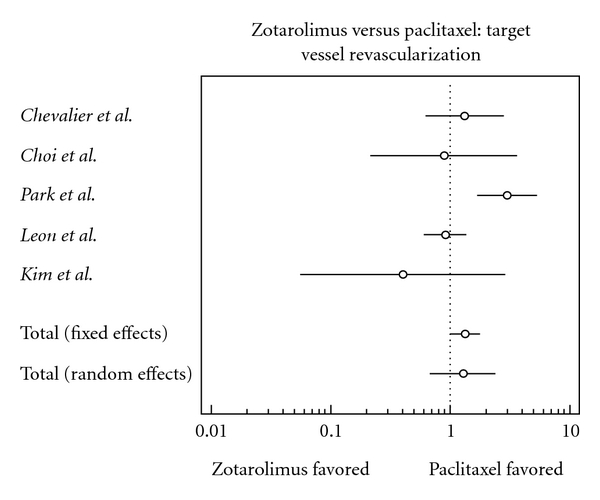
Forest plot comparing risk of target vessel revascularization.

**Figure 8:**
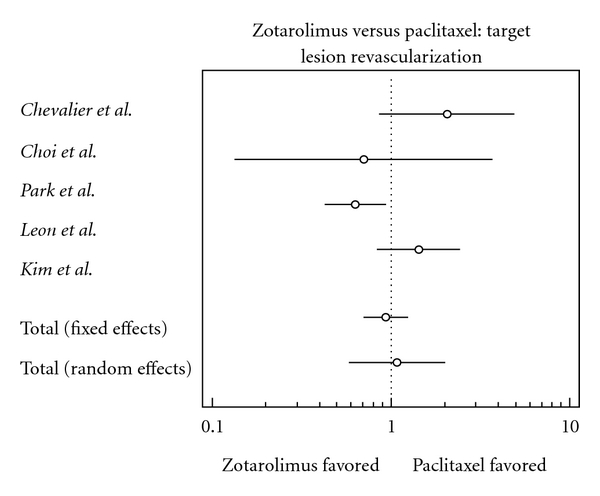
Forest plot comparing risk of target lesion revascularization.

The characteristics of each individual trial had no significant differences within studies (Table 2). Results of the meta-analysis are shown in Figures 2, 3, 4, 5, 6, 7, and 8. There were no significant differences in patient demographics between both groups.

### 3.2. Myocardial Infarction

There was a significant decrease in risk of myocardial infarction in the zotarolimus group (odds ratio, 0.250, confidence interval, 0.160 to 0.392).

### 3.3. Major Adverse Cardiac Events

There was a slightly lower risk of major adverse cardiac events in the zotarolimus group; this finding, however, is statistically insignificant (odds ratio, 0.813, confidence interval, 0.656 to 1.007).

### 3.4. Cardiac Death

There was a slightly lower risk of cardiac death in the zotarolimus group; this finding, however, is statistically insignificant (odds ratio, 0.817, confidence interval, 0.359 to 1.857).

### 3.5. All-Cause Death

There was a slightly lower risk of all-cause death in the zotarolimus group; this finding, however, is statistically insignificant (odds ratio, 0.820, confidence interval, 0.443 to 1.516).

### 3.6. Stent Thrombosis

There was a slightly higher risk of stent thrombosis in the zotarolimus group; this finding, however, is statistically insignificant (odds ratio, 1.174, confidence interval, 0.604 to 2.280).

### 3.7. Target Vessel Revascularization

There was a significantly higher risk of target vessel revascularization in the zotarolimus group (odds ratio, 1.336, confidence interval, 1.003 to 1.778).

### 3.8. Target Lesion Revascularization

Risk of target lesion was lower in the zotarolimus group; this finding, however, is statistically insignificant (odds ratio, 0.936, confidence interval 0.702 to 1.247).

## 4. Discussion

This meta-analysis shows that zotarolimus-eluting stents may not differ with respect to outcomes studied here. Other studies have had similar results with these particular outcomes when comparing zotarolimus- and paclitaxel-eluting stents [3–5]. A two-year follow-up study by Cicek et al., however, did find a statistically significant decrease in MACE, coronary artery bypass graft, and Q wave MI, associated with zotarolimus-eluting stents when compared to paclitaxel-eluting stents. No major differences were reported by this study for all-cause death, target vessel revascularization, and non-target-vessel revascularization [7]. This study was nonrandomized and had a small number of patients; so the true value of these findings is questionable. Where the two stent varieties do seem to differ is in late loss, an end point not included in this 12-month outcome meta-analysis. Zotarolimus-eluting stents have been documented to demonstrate greater late loss than paclitaxel-eluting stents [2, 3, 8]. This study, however, did not find an increase in stent thrombosis with zotarolimus-eluting stents.

Drug-eluting stents have significantly reduced the rate of restenosis when compared to bare metal stents [9] and have assumed a larger role in the management of STEMI as primary percutaneous coronary intervention has become standard of care. When compared to bare metal stents, drug-eluting stents have shown greater 1-year event-free survival rates [10–12] and similar safety profiles [13, 14] in STEMI patients. Sirolimus- and paclitaxel-eluting stents have been used with success for primary PCI in standard STEMI cases as well as those complicated by anatomical variations [15].

The ENDEAVOR IV trial studied the cost-effectiveness of zotarolimus-eluting stents and concluded that cost-effectiveness was similar to that of paclitaxel-eluting stents. Analysis took into account quality-adjusted survival, medical costs, and relative cost-cost effectiveness [16].

While zotarolimus-eluting stents have been associated with greater in-stent late loss when compared to paclitaxel-eluting stents, this may not have much clinical impact as studies have noted that low mean values of in-stent late loss are not associated with the risk of stent thrombosis [17]. 

Limitations of this meta-analysis include those inherent to all such analyses such as pooling of data from studies which may have slightly differing designs and heterogeneity. It should also be noted that ZoMaxx I included only 9-month follow-up data which was included in this study. As with any meta-analysis, publication and selection bias may have impacted results of this analysis.

## 5. Conclusion

The zotarolimus-eluting stents offer a safe option when selecting a drug-eluting stent. When compared to paclitaxel-eluting stents, zotarolimus eluting stents are associated with a significantly lower risk of myocardial infarction while being associated with a significantly higher risk of need for target vessel revascularization. Additionally, studies have shown increased late loss in zotarolimus-eluting stents when compared to paclitaxel-eluting stents. This increased late loss should be kept in mind when zotarolimus-eluting stents are being considered. Comparison of 12-month outcomes does not seem to warrant the use of either stent over the other, particularly keeping in mind the absence of any advantage afforded by one stent in regards to cost-effectiveness.

##  Financial Disclosures

There are no financial disclosures to be made regarding this manuscript. All the authors have contributed to the manuscript and approved its submission. This manuscript has not been submitted to any other journal or has not been presented in any other form.

## Figures and Tables

**Table 1:** Summary of heterogeneity analysis.

	*Q*	*dF*	*P*-value	Result
MI	1.6724	3	.6431	Homogenous
MACE	3.1045	2	.2118	Homogenous
Cardiac death	0.8357	3	.8357	Homogenous
All-cause death	3.1045	2	.2118	Homogenous
Stent thrombosis	0.2858	3	.9627	Homogenous
Target vessel revascularization	6.7677	3	.0797	Homogenous
Target lesion revascularization	5.8443	2	.0538	Homogenous

*dF*: degrees of freedom.

**Table 2:** Baseline characteristics of patients in included studies.

	Chevalier et al.	Choi et al.	Kim et al.	Park et al.	Leon et al.
Number studied					
PES	197	153	105	884	775
ZES	199	86	47	883	773
Age					
PES	63 +/− 11	61.54 +/− 12.22	63 +/− 9	62.0 +/− 9.6	63.6 +/− 11.0
Zes	63 +/− 10	60.24 +/− 11.76	59 +/− 12	61.7 +/− 9.3	63.5 +/− 11.1
Gender (male)					
PES	77%	100 (65.4%)	76 (72.4%)	582 (65.8%)	531 (68.5%)
ZES	75%	69 (80.2%)	37 (78.7%)	586 (66.4%)	517 (66.9%)
Vessel location (LAD)					
PES	40%	72 (47.1%)	45 (42.9%)	611 (50.7%)	321 (41.5%)
ZES	48%	45 (52.3%)	30 (63.8%)	622 (52.3%)	326 (42.2%)
Vessel location (LCx)					
PES	19%	29 (19.0%)	15 (14.2%)	253 (21.0)	202 (26.1%)
ZES	24%	10 (11.6%)	2 (4.3%)	252 (21.2%)	208 (26.9%)
Vessel location (RCA)					
PES	41%	52 (34.0%)	45 (42.9)	340 (28.2%)	251 (32.4%)
ZES	28%	31 (36.0%)	15 (31.9%)	316 (26.6)	238 (30.8%)
Diabetes mellitus					
PES	26%	104 (68.0%)	23 (21.9%)	245 (27.7%)	236 (30.5%)
ZES	22%	64 (74.4%)	12 (25.5%)	268 (30.4%)	241 (31.2%)
Hypertension					
PES	67%	86 (56.2%)	50 (47.6%)	540 (61.1%)	640 (82.6%)
ZES	69%	53 (61.6%)	20 (43.5%)	552 (62.5%)	614 (79.4%)
Hyperlipidemia					
PES	72%	104 (68.2%)	43 (41.3%)	446 (50.5%)	657 (84.8%)
ZES	78%	57 (66.3%)	18 (39.1%)	466 (52.8%)	629 (81.4)

PES: paclitaxel-eluting stent; ZES: zotarolimus-eluting stent; LAD: left anterior descending coronary artery; LCx: left circumflex coronary artery; RCA: right coronary artery.

## Abbreviations

CABG: Coronary artery bypass graft
MI: Myocardial infarction
MACE: Major adverse cardiac events
STEMI: ST-elevation myocardial infarction
PCI: Percutaneous coronary intervention.
